# Effect of the COVID-19 Lockdown on Pediatric Bronchial Asthma Patients in Saudi Arabia: A Comparative Study

**DOI:** 10.7759/cureus.75774

**Published:** 2024-12-15

**Authors:** Manal Bawazeer, Aljowhara F Saad, Bushra S Aljuhani, Sarah S Mawlaalduwilah, Akaber M Aljoudi, Raneem A Gomawi, Nazish Masud

**Affiliations:** 1 Department of Pediatrics, King Abdullah Specialized Children's Hospital, Riyadh, SAU; 2 Research Unit, Department of Medical Education, King Saud Bin Abdulaziz University for Health Sciences, Riyadh, SAU; 3 Research Office, King Abdullah International Medical Research Center, Riyadh, SAU; 4 College of Medicine, King Saud Bin Abdulaziz University for Health Sciences, Riyadh, SAU; 5 Research Unit, College of Medicine, King Saud Bin Abdulaziz University for Health Sciences, Riyadh, SAU

**Keywords:** children, covid-19, lockdown, moderate asthma, pandemic, severe asthma

## Abstract

Background: The lockdowns imposed during the COVID-19 pandemic and social distancing measures may have decreased traffic and air pollution, which may contribute to reducing asthma exacerbation. However, there is not enough information about the relationship between asthma and COVID-19 lockdown, especially in children. Therefore, the aim of this study is to identify the effects of the COVID-19 lockdown on pediatric patients with moderate to severe bronchial asthma.

Methods: This is a retrospective cross-sectional analytical study of pediatric patients with moderate to severe asthma who came to King Abdullah Specialist Children’s Hospital (KASCH) in Riyadh, Saudi Arabia. The study was conducted for a period of 14 months from March 2019 to May 2020, using the charts of children aged 3 to 14 years. We investigated changes in the severity of asthma using indirect parameters including hospital visits, ER visits, and changes in medications used before and during the COVID-19 lockdown.

Results: A total of 343 asthmatic patients aged mean±SD of 8±3 years were included in the study. More than half 233 (68%) of them were male. The number of patients admitted to the hospital in 2019 was 46 patients (85%), while in 2020, it was only 17 patients (32%). In 2020, the usage of oral steroids has been decreased from 96 (28%) to 50 (15%). The number of people using the leukotriene inhibitor reduced from 171 in 2019 to 162 in 2020. The ER mean visit was 1.6±1.3 in 2019; however, ER visits in 2020 were 0.6 ±0.7 showing a considerable reduction in the ER visits (p < 0.001).

Conclusion: The COVID-19 lockdown had a positive impact on asthma patients, with our study showing a significant reduction in ER visits, hospitalizations, and the use of oral steroids between March-May 2019 and 2020, suggesting lower asthma severity. However, a holistic approach is needed post-pandemic to improve asthma management, including increased awareness, better healthcare access, and reduction of environmental triggers to promote better control and overall well-being

## Introduction

Nowadays, respiratory health is a major concern worldwide [1]. There are many factors that increase the sensitivity of the lungs and cause asthma [1]. Asthma is one of the most common chronic respiratory diseases among children [2]. It is characterized by a reversible bronchial obstruction that can be mild, moderate, or severe [3]. The obstruction can be triggered by allergens, smoking, and air pollution [4]. In 2016, around 339 million people were affected with asthma worldwide and the numbers are expected to dramatically increase by 2025 [3,4]. The recent coronavirus disease 2019 (COVID-19) lockdown improved the environmental quality [5]. It showed a significant reduction of air pollutants that may have positive effects on patients with moderate to severe asthma [5].

The COVID-19 pandemic is a respiratory condition that has a high incidence rate [6,7]. This infectious disease affects people of all ages [7]. The severity of this infection varies significantly between people [7]. Some people have an asymptomatic infection [7]. Others may develop severe disease that leads to death, especially those who have chronic disorders [7]. Since there was no evidence that antiviral medicines or vaccines were effective against COVID-19 at the time, the most effective way to reduce the global spread of the virus was by restricting human mobility and activity [7,8]. Therefore, the whole world adopted the lockdown and curfew to control the virus spreading [7,8]. As all human activity shut off, nature took the opportunity and showed great improvement in the quality of air and water [8].

Air pollution is one of the major concerns for children with asthma [5,8]. The persistence of air pollution in urban areas captured a great deal of attention in affecting patients with asthma for several years [8,9]. A recent study conducted in Pakistan in 2022 found that air pollutants were significantly high, posing potentially harmful effects on air quality and public health [10]. Similarly, a 2018 study showed that exposure to pollutants such as nitrogen dioxide, ozone, and sulfur dioxide was associated with negative asthma outcomes [11]. The quality of the air has an impact on asthma aggravation that leads to higher urgent hospital visits and admissions [12]. It also increases the number of medications used by the patients due to the triggered bronchial hyper-responsiveness when exposed to polluted air [12]. Before the COVID-19 pandemic, 8% of deaths around the world were caused by air pollution according to the World Health Organization (WHO) report [13]. Although COVID-19 has a severe negative impact on human health and the world economy, it has a great positive impact on the global environment [13]. Patients with asthma were thought to be at higher risk of developing serious diseases if infected with COVID-19 [14]. However, recent studies showed that the number of people with asthma affected by COVID-19 was much lower than the general population [15,16]. Also, the urgent doctor visits of both adult and children patients with moderate to severe asthma were remarkably declined [16]. These findings are very important because usually, patients with bronchial inflammatory diseases have an increased risk of viral infections [15]. An explanation for these observations could be due to the pharmacological mechanisms of drugs these patients use [17]. Studies suggest that biological agents that are used to treat asthma could prevent the infection and the severity of COVID-19 [17]. Another important factor that may have caused the reduction in emergency visits of patients with asthma is the reduced air pollution and improved environmental quality after the COVID-19 lockdown [17].

Asthma and COVID-19 are respiratory diseases that could have a huge impact on health and the economy globally [1,16]. Many studies have shown the serious effects of COVID-19 on asthma patients [17]. Still, the effect of the COVID-19 pandemic lockdown on patterns of presentation of bronchial asthma is not completely understood, especially in children [15,16]. Therefore, the study aimed to identify the effects of the COVID-19 lockdown on pediatric patients with moderate to severe bronchial asthma.

## Materials and methods

This was a retrospective cross-sectional analytical pre- and post-study conducted for a period of 14 months from March 2019 to May 2020 at King Abdullah Specialist Children’s Hospital (KASCH) in Riyadh, Saudi Arabia, which is the first specialist Children’s Hospital in the Kingdom of Saudi Arabia, occupying 192,000 m^2^, over 10 levels. The hospital has a four-floor podium level accommodating the Emergency Department and state-of-the-art DayCare, Diagnostic and Treatment Departments. The hospital has 60 beds in the emergency department for Pediatric Emergency and Trauma. The electronic medical records of the patients were retrieved for the specified period of time, using the BestCare® patient file management system already available in the hospital.

The study utilized secondary data from children (3 to 14 years) with moderate to severe Asthma. All children with the diagnosis of moderate to severe asthma seen in the emergency department (ED), pediatric Allergy and Immunology, General Pediatrics and pediatric Pulmonology at the hospital were included in the study.

Identification of study participants

The inclusion criteria included pediatric patients (male and female) from 3 to 14 years old who have moderate to severe asthma symptoms. The patients who were seen in the months of March to September 2019 and March to September 2022 were included if they were seen at two points in time during the same months in each year. This inclusion based on the months was fixed in order to make pre- and post-comparisons. This strict inclusion leads to the exclusion of many patients from the final data analysis. Additionally, patients with comorbid conditions were excluded to decrease the over-reporting of ER visits. Using the consecutive sampling technique, the patient's records showed that 2120 charts met the inclusion criteria. However, 1777 of them were excluded for three main reasons. Two patients were older than 14 years old, 77 patients were less than three years old, and 1698 failed to follow up at two points in time during the specified study period. Therefore, the final number included in the analysis was 343.

Data collection process

The data was collected using the BestCare system placed in the hospital to access the patient records. The research team members extracted the data using Microsoft Excel. The main variables included demographic variables-age, gender, height and weight, asthma history, medications used before and during the COVID-19 lockdown, and hospital and ER visits before and during the COVID-19 lockdown. 

Data analysis

The data was collected in MS Excel and transferred to IBM SPSS Statistics for Windows, Version 24 (Released 2016; IBM Corp., Armonk, New York, United States) for statistical analysis. Results are presented in tables and figures. Categorical variables were expressed as frequencies and percentages like gender, age categories, and asthma medication. Mean, SD, and median were used for continuous variables like dosage of medicine, age in years, height, weight, etc. All the comparisons were done for the years 2019 and 2020 and the same patient was assessed at two points in time. Therefore, the Wilcoxon signed-rank test and McNemar test were used to report before and after comparison for continuous and categorical variables respectively. The p-value was kept at <0.05 to be considered significant for the tests applied.

Ethical considerations

The study was conducted after approval from the ethical review committee of King Abdullah International Medical Research Center (KAIMRC) with memo no: RYD-20-419812-152389. As the study is a chart review, informed consent was not required from the patients. However, official approval was taken for accessing the medical record. Participants’ confidentiality and anonymity were strictly observed throughout the study by using serial numbers for each subject and restricting data access to the investigators only.

## Results

**Figure 1:**
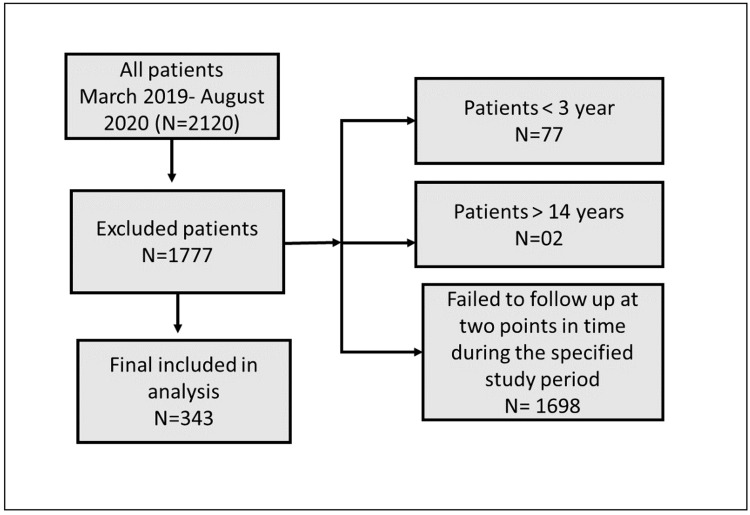
Patients included in the final analysis.

The flowchart in Figure 1 illustrates that out of a total of 2,120 patients initially considered, 1,777 were excluded based on specific criteria. The exclusion criteria were patients younger than three years (77 patients), patients older than 14 years (two patients), and patients who failed to follow up at two points in time during the specified study period (1,698 patients). After applying these exclusion criteria, 343 patients were included in the final analysis.

Table 1 shows that the mean age of patients is 8±3 years while the mean of asthma diagnosis age is 5.5 ± 2.3 years. Fifteen percent of patients have the highest growth percentile which is >97%. Most patients were diagnosed in the general pediatrics department (78% of patients).

**Table 1:** Summary of the patient profile. Data of continuous variables presented as mean± std. Categorical variables presented as count (n) and frequencies (%).

Variables	Unit/Category	Mean ± SD/ N (%)
Age	Years	8±3
Asthma diagnosis	Years	5.5±2.3
Height	cm	129.1±21.4
Weight	kg	33.4±17.4
Gender	Male	233 (68%)
	Female	110 (32%)
Growth percentile	<3rd	44 (13%)
	3rd-5th	5 (2%)
	5th-10th	19 (6%)
	10th-25th	43 (13%)
	25th-50th	45 (14%)
	50th-75th	43 (13%)
	75th-85th	21 (6%)
	85th-90th	18 (5%)
	90th-95th	22 (7%)
	95th-97th	25 (8%)
	>97th	49 (15%)
Presence of other allergies	No	230 (67%)
	Yes	113 (33%)
Diagnosis department	General Pediatrics	266 (78%)
	Pediatric Allergy ＆ Immunology	18 (5%)
	Pediatric Pulmonology	29 (9%)
	Others	30 (9%)

Table 2 shows the patients' admission and usage of medication in 2019 vs 2020. The study observed that the number of hospital admissions and the usage of oral steroids had been significantly reduced during the quarantine. The number of patients admitted to the hospital in 2019 was 46 patients (85%), while in 2020, it was only 17 patients (32%). In 2020, the usage of oral steroids has been decreased from 96 (28%) to 50 (15%).

**Table 2:** Patient admission and usage of medication in 2019 vs 2020. Data has been presented as count (n) and frequencies (%).

Variables	2019		2020	
	Category	N (%)	Category	N (%)
Hospital admission	No	8 (15)	No	37 (69)
	Yes	46 (85)	Yes	17 (32)
Beta2 agonist	No	28 (8)	No	31 (9)
	Yes	314 (92)	Yes	311 (91)
Steroid inhaler	No	71 (21)	No	73 (21)
	Yes	271 (79)	Yes	269 (79)
Leukotriene inhibitors	No	170 (50)	No	179 (53)
	Yes	171 (50)	Yes	162 (48)
Oral steroids	No	243 (72)	No	291 (85)
	Yes	96 (28)	Yes	50 (15)

**Figure 2:**
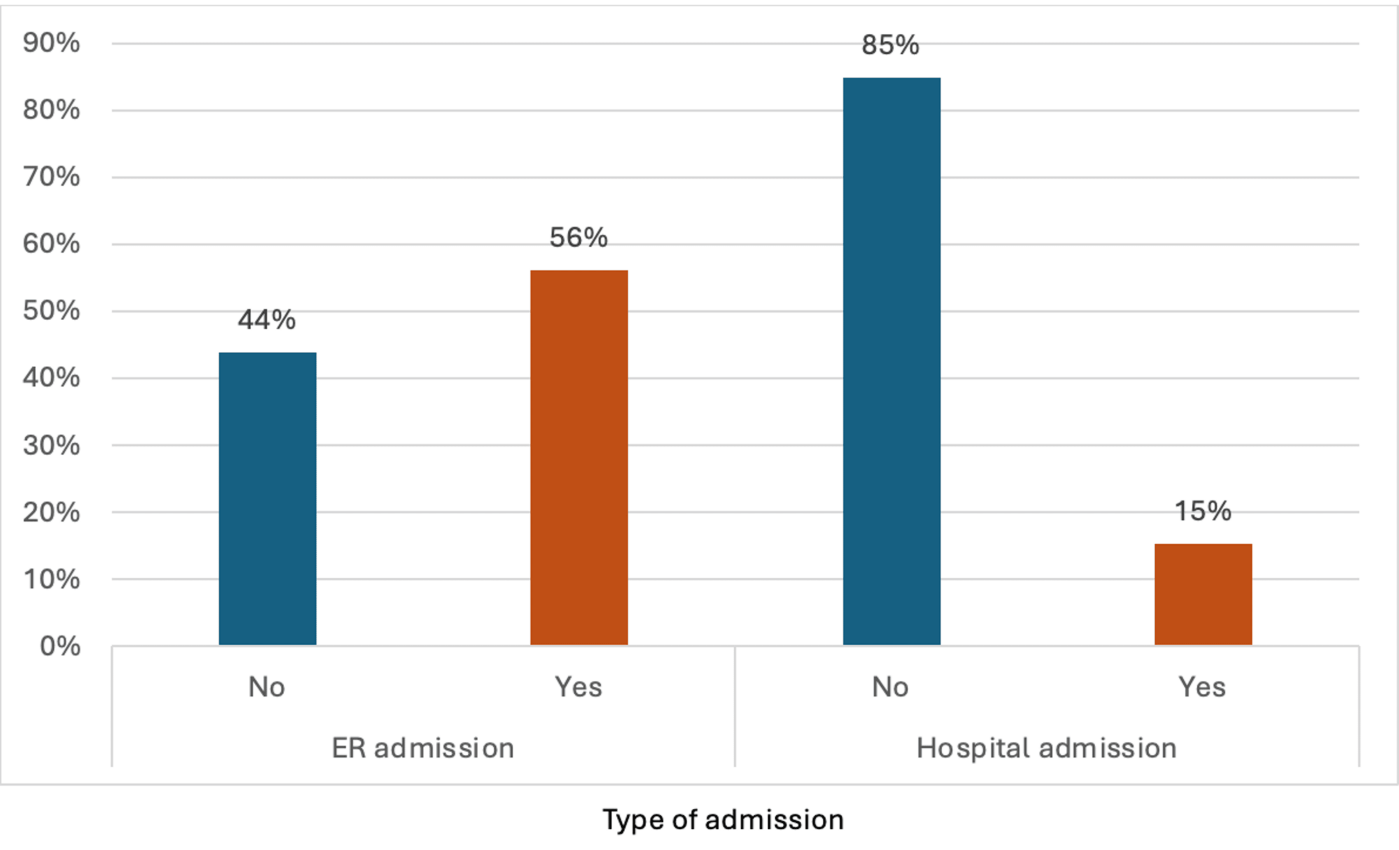
Total hospital admissions and emergency room (ER) visits among patients.

Figure 2 illustrates ER and hospital admissions for pediatric asthma patients in 2019. For ER admissions, a higher percentage of patients (56%) were admitted compared to those who were not admitted (44%). In contrast, the distribution for hospital admissions shows that the majority of patients (85%) did not require hospital admission, while only 15% were admitted to the hospital.

Table 3 shows a non-parametric version of the paired t-test. Many of the variables were not normally distributed, so a non-parametric Wilcoxon signed-rank test was used. The number of people using the leukotriene inhibitor reduced from 171 in 2019 to 162 in 2020; however, the mean dosage increased from (4.6 ±1.0) to (4.9 ±1.0).

**Table 3:** Comparison of medication use among patients during 2019 vs 2020. Data is summarized using the mean ± standard deviation (SD) and the median with interquartile range (Q1-Q3). * Wilcoxon signed-ranks test significant at p<0.05

Medication Use	N	Mean ± SD	Median(Q1-Q3)	p-value
Dose of Beta2 agonist 2019	262	2.7±1.0	2(2-4)	0.06
Dose of Beta2 agonist 2020	280	2.6±0.9	2(2-4)	
Frequency of Beta2 agonist 2019	314	4.0 ±1.5	4(4-4)	0.38
Frequency of Beta2 agonist 2020	311	4.0 ±1.3	4(4-4)	
Dose of steroid inhaler 2019	272	1.8 ±0.5	2(2-2)	0.93
Dose of steroid inhaler 2020	267	1.8 ±0.6	2(2-2)	
Frequency of steroid inhaler 2019	271	2.0 ±0.3	2(2-2)	0.84
Frequency of steroid inhaler 2020	269	2.0 ±0.3	2(2-2)	
Dose of leukotriene inhibitors 2019	171	4.6 ±1.0	5(4-5)	<0.001*
Dose of leukotriene inhibitors 2020	163	4.9 ±1.0	5(5-5)	
Frequency of leukotriene inhibitors 2019	171	1.0 ±0.1	1(1-1)	1
Frequency of leukotriene inhibitors 2020	162	1.0 ±0.0	1(1-1)	
Dose of oral steroids 2019	97	13.9 ±6.5	12(10-16)	0.38
Dose of oral steroids 2020	50	14.3 ±7.8	14(8.8-16.5)	

**Figure 3:**
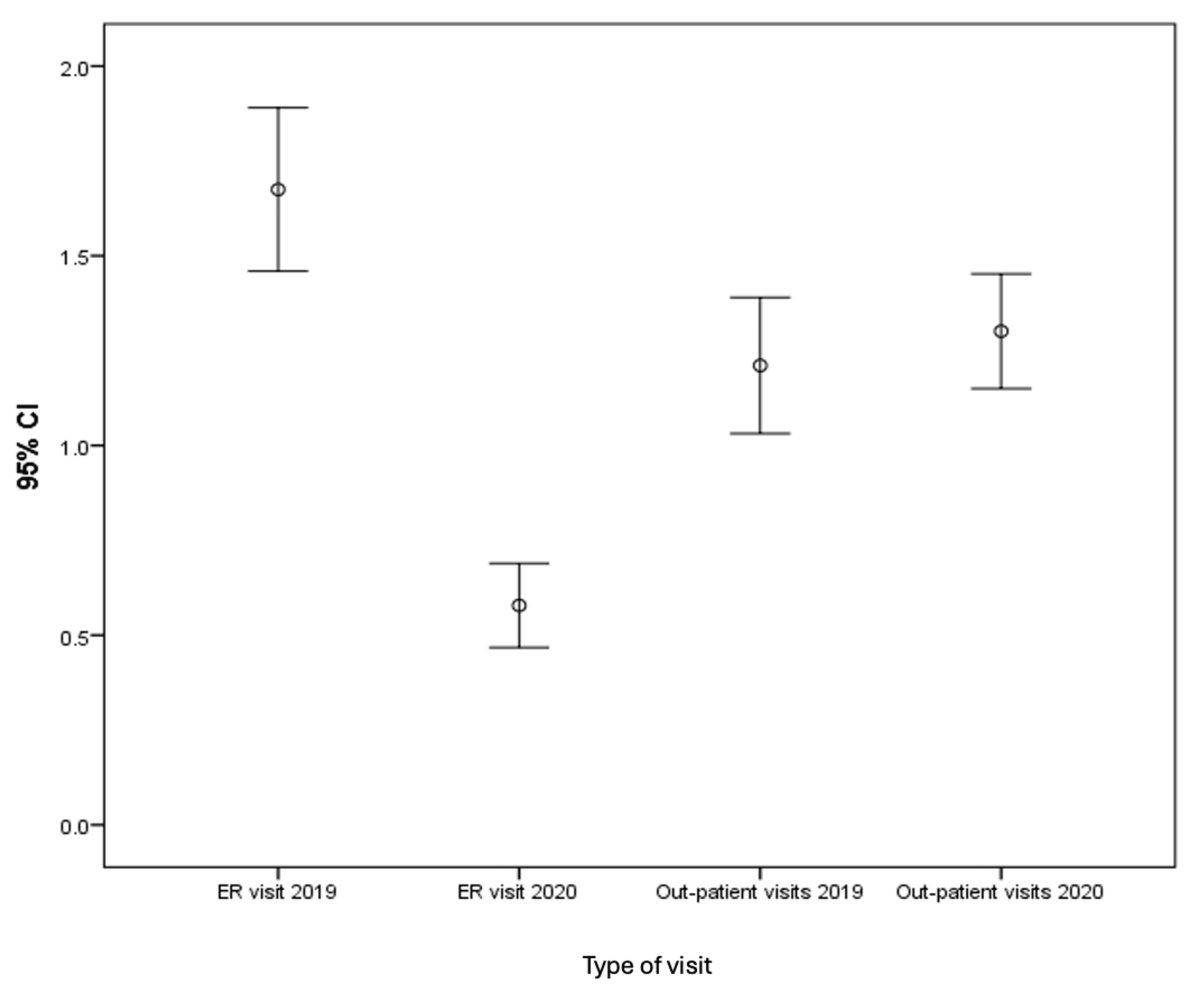
Error bar chart of ER and outpatient visits in 2019 and 2020. The error bars are used to represent the range of values

Figure 3 shows a 95% confidence interval of ER visits in 2019 and 2020 as well as outpatient visits in 2019 and 2020. ER visits in 2019 mean (1.6 ±1.3); however, ER visits in 2020 (0.6 ±0.7) suggested significant improvement in controlling asthma (P < 0.001).

The results from Table 4, based on the McNemar test, show that 36 patients (97.3%) who were admitted to the hospital in 2019 were not admitted in 2020 with p<0.001. This indicates that there was a significant reduction in hospital admissions in 2020. Besides, there was no significant reduction in medication use, except for oral steroids, where about 73 patients (25.2%) used oral steroids in 2020 (Table 4).

**Table 4:** McNemar test for hospital admission and medication usage in 2019 vs 2020. Data has been presented as count (n) and frequencies (%) *McNemar test applied, significant at p<0.05.

Treatment	2019 (N, %)	2020 (N, %)	p-value
HA - No	1 (2.7%)	7 (41.20%)	<0.001*
HA - Yes	36 (97.3%)	10 (58.80%)	
Beta2 agonist - No	4 (12.90%)	24 (7.70%)	0.78
Beta2 agonist - Yes	27 (87.10%)	286 (92.30%)	
Steroid inhaler - No	34 (46.60%)	37 (13.80%)	0.91
Steroid inhaler - Yes	39 (53.40%)	231 (86.20%)	
Leukotriene inhibitors - No	132 (74.60%)	38 (23.50%)	0.51
Leukotriene inhibitors - Yes	45 (25.40%)	124 (76.50%)	
Oral steroids - No	217 (74.80%)	25 (53.20%)	<0.001*
Oral steroids - Yes	73 (25.20%)	22 (46.80%)	

## Discussion

This study aimed to assess the effect of the COVID-19 lockdown on the usage of asthma medications and the risk of exacerbation of asthma by the number of emergency department visits and hospital admissions due to flare-ups among pediatric patients. The data show that 56% of patients visited the ER, and 15% were hospitalized during the study period, with a significant reduction in both hospital admissions and the use of oral steroids in 2020 compared to 2019. Hospital admissions dropped from 85% in 2019 to 32% in 2020, while oral steroid usage decreased from 28% to 15%. This reduction is attributed to changes in asthma exacerbation patterns and patients' perceptions of their condition, which led to a considerable decrease in in-person visits during the pandemic lockdown. Since oral steroids are used to control moderate to severe asthma attacks, this indirectly indicates a reduction in the overall severity of asthma among pediatric patients [18]. In addition, the reduction in ER visits and hospital admissions could be due to a drop in asthma exacerbation during the COVID-19 lockdown. These observations might be attributed to the reduction in atmospheric pollution, changes in healthcare utilization behavior, or improved parental management of asthma attacks. 

The results reveal that there was a slight reduction in the number of patients using leukotriene inhibitors. Yet, despite fewer patients using leukotriene inhibitors, the mean dosage increased significantly from 4.6 to 4.9, suggesting an adjustment in treatment intensity. However, the use of Beta2 agonists, inhaled steroids, and leukotriene inhibitors remained relatively stable between 2019 and 2020. The Wilcoxon signed-rank test confirmed significant changes in leukotriene inhibitor dosages (p < 0.001), but no significant differences were observed in the dosages and frequencies of Beta2 agonists or inhaled steroids between the two years. Furthermore, the McNemar test results demonstrated a significant reduction in hospital admissions and oral steroid use in 2020 compared to 2019 (p < 0.001), but no significant reductions were noted for other medications.

The overall improvement in asthma control during 2020, reflected by the decrease in ER visits (from 1.6 ±1.3 in 2019 to 0.6 ±0.7 in 2020) and the reduced hospital admissions, suggests that external factors during quarantine, such as fewer respiratory infections and reduced exposure to allergens, may have positively influenced asthma management. A study showed that the use of systemic corticosteroids in adults and children with acute asthma decreased the rate of hospital admissions [19]. Kenyon and colleagues conducted a study to assess asthma-related emergency visits in children’s hospitals during the COVID-19 pandemic [20]. Their findings demonstrate a marked drop in emergency utilization for asthma of all severities [20]. A study from Saudi Arabia (KSA) found that the number of hospitalizations among adults with asthma was significantly reduced during the COVID-19 lockdown [17]. Another study from Nigeria observed that social distancing and public health measures led to the avoidance or postponement of visits to healthcare facilities, resulting in missed vital check-ups and preventive care, which ultimately contributed to an increase in respiratory morbidities among pediatric asthma patients during the COVID-19 lockdown [21].

One of the main limitations of the study is its retrospective design, which relied on a secondary dataset. Since many patients were not seen during one of the specific time periods in our study, they could not be assessed at two separate points in time. As a result, the sample size was relatively small after excluding these patients from the data. Moreover, when extracting information from a secondary data set, it was difficult to know if the patient was taking a medication (beta-agonist and bronchodilators) as an inhaler or nebulizer and then deciding the dose. This could be a reason for not seeing a significant change in patients’ dose or frequency of some medications in our study. In order to control the effect of season, weather, and other environmental effects on asthma, the selection was strictly based on the corresponding months in two years which is one of the strengths of our study design. A major strength of this study is that we included all asthma pediatric patients in more than three different departments and tracked each one at two points in time. Moreover, the information and knowledge gained from this study will contribute positively to knowing more about bronchial asthma control where the effects of the environment and COVID-19 social distancing measures might have an impact. 

We recommend that future studies be done with a larger sample and in multiple centers in different regions within the country in order to show a better picture of the effects of a devastating pandemic like COVID-19 among pediatric patients in other regions. Furthermore, we recommend that some of the secondary data that is reported in the hospital be improved for future studies to illustrate the severity of asthma in each pediatric patient because we had to assess asthma severity in these patients with indirect indicators, and this was beyond the scope of our research.

## Conclusions

The COVID-19 lockdown had a positive impact on patients with asthma. Our study shows a significant reduction in both ER visits and hospitalizations between the periods of March-May 2019 and 2020. Additionally, the use of oral steroids to manage asthma exacerbations decreased, suggesting that asthma severity was lower during the lockdown. However, there is a need for a holistic approach to improving the quality of life for asthmatic patients post-pandemic. This approach should include increased awareness and education, improved access to healthcare, and the reduction of environmental triggers all of which promote better asthma control and overall well-being

## References

[1] (2018). National Heart, Lung, and Blood Institute (NHLBI). https://www.nih.gov/about-nih/what-we-do/nih-almanac/national-heart-lung-blood-institute-nhlbi.

[2] Ferrante G, La Grutta S (2018). The burden of pediatric asthma. Front Pediatr.

[3] Busse W (2007). Expert Panel Report 3: Guidelines for the Diagnosis and Management of Asthma. https://www.ncbi.nlm.nih.gov/books/NBK7232/.

[4] (2022). The Global Asthma Report 2022. https://www.globalasthmanetwork.org/Global_Asthma_Report_2022.pdf.

[5] Gasana J, Dillikar D, Mendy A, Forno E, Ramos Vieira E (2012). Motor vehicle air pollution and asthma in children: a meta-analysis. Environ Res.

[6] Rothan HA, Byrareddy SN (2020). The epidemiology and pathogenesis of coronavirus disease (COVID-19) outbreak. J Autoimmun.

[7] Nussbaumer-Streit B, Mayr V, Dobrescu AI (2020). Quarantine alone or in combination with other public health measures to control COVID-19: a rapid review. Cochrane Database Syst Rev.

[8] Arora S, Bhaukhandi KD, Mishra PK (2020). Coronavirus lockdown helped the environment to bounce back. Sci Total Environ.

[9] Schwartz J (1994). What are people dying of on high air pollution days?. Environ Res.

[10] Zahra SI, Iqbal MJ, Ashraf S, Aslam A, Ibrahim M, Yamin M, Vithanage M (2022). Comparison of ambient air quality among industrial and residential areas of a typical south Asian city. Atmosphere.

[11] Burbank AJ, Peden DB (2018). Assessing the impact of air pollution on childhood asthma morbidity: how, when, and what to do. Curr Opin Allergy Clin Immunol.

[12] Masoumi K, Haddadzadeh Shoushtari M, Forouzan A, Asgari Darian A, Dastoorpoor M, Ebrahimzadeh P, Aghababaeian H (2017). Rainfall-associated bronchospasm epidemics: the epidemiological effects of air pollutants and weather variables. Can Respir J.

[13] (2018). WHO: 9 Out Of 10 People Worldwide Breathe Polluted Air, But More Countries Are Taking Action. https://www.who.int/news/item/02-05-2018-9-out-of-10-people-worldwide-breathe-polluted-air-but-more-countries-are-taking-action.

[14] (2020). Coronavirus Disease 2019 (COVID-19) [Internet]. Centers for Disease Control and Prevention. https://www.cdc.gov/covid/index.html.

[15] Caminati M, Lombardi C, Micheletto C (2020). Asthmatic patients in COVID-19 outbreak: few cases despite many cases. J Allergy Clin Immunol.

[16] Gupta A, Bush A, Nagakumar P (2020). Asthma in children during the COVID-19 pandemic: lessons from lockdown and future directions for management. Lancet Respir Med.

[17] Ayaz KM, Rajkumar R, Basma AG (2021). The effects of the COVID-19 lockdown on severe asthma in patients taking biologic therapy and air pollution in Riyadh. Ann Thorac Med.

[18] Rowe BH, Spooner C, Ducharme FM, Bretzlaff JA, Bota GW (2000). Early emergency department treatment of acute asthma with systemic corticosteroids. Cochrane Database Syst Rev.

[19] Alangari AA (2014). Corticosteroids in the treatment of acute asthma. Ann Thorac Med.

[20] Kenyon CC, Hill DA, Henrickson SE, Bryant-Stephens TC, Zorc JJ (2020). Initial effects of the COVID-19 pandemic on pediatric asthma emergency department utilization. J Allergy Clin Immunol Pract.

[21] Isezuo KO, Sani UM, Waziri UM (2024). Patterns and outcomes of Emergency Pediatric Unit admissions in Usmanu Danfodiyo University Teaching Hospital in Sokoto State, Nigeria: a five-year review. Pyramid J Med.

